# Characterisation of terrestrial acidophilic archaeal ammonia oxidisers and their inhibition and stimulation by organic compounds

**DOI:** 10.1111/1574-6941.12353

**Published:** 2014-07-31

**Authors:** Laura E Lehtovirta-Morley, Chaorong Ge, Jenna Ross, Huaiying Yao, Graeme W Nicol, James I Prosser

**Affiliations:** 1 Institute of Biological and Environmental Sciences, University of AberdeenAberdeen, UK; 2 The First Affiliated Hospital, Zhejiang UniversityHangzhou, China; 3 Institute of Urban Environment, Chinese Academy of SciencesXiamen, China

**Keywords:** ammonia-oxidising archaea, acidic soil, low pH, mixotrophy, autotrophy, nitrification

## Abstract

Autotrophic ammonia oxidation is performed by two distinct groups of microorganisms: ammonia-oxidising archaea (AOA) and ammonia-oxidising bacteria (AOB). AOA outnumber their bacterial counterparts in many soils, at times by several orders of magnitude, but relatively little is known of their physiology due to the lack of cultivated isolates. Although a number of AOA have been cultivated from soil, *Nitrososphaera viennensis* was the sole terrestrial AOA in pure culture and requires pyruvate for growth in the laboratory. Here, we describe isolation in pure culture and characterisation of two acidophilic terrestrial AOA representing the *Candidatus* genus *Nitrosotalea* and their responses to organic acids. Interestingly, despite their close phylogenetic relatedness, the two *Nitrosotalea* strains exhibited differences in physiological features, including specific growth rate, temperature preference and to an extent, response to organic compounds. In contrast to *N. viennensis*, both *Nitrosotalea* isolates were inhibited by pyruvate but their growth yield increased in the presence of oxaloacetate. This study demonstrates physiological diversity within AOA species and between different AOA genera. Different preferences for organic compounds potentially influence the favoured localisation of ammonia oxidisers within the soil and the structure of ammonia-oxidising communities in terrestrial ecosystems.

## Introduction

Ammonia oxidation is the first and rate-limiting step of nitrification. While ammonia-oxidising bacteria (AOB) have been cultivated for more than a century, the first ammonia-oxidising archaeon (AOA) *Nitrosopumilus maritimus* was not isolated until 2005 (Könneke *et al*., 2005). Since then, 27 different cultivated AOA strains have been reported (Supporting information, Table S1), spanning six proposed genera and originating from a diverse range of aquatic and terrestrial habitats. However, only two pure cultures have been obtained so far, and *Nitrososphaera viennensis* is the only representative from soil (Tourna *et al.*, 2011). This lack of pure cultures currently limits studies of comparative physiology within AOA and between AOA and AOB.

Ammonia oxidisers, whether bacterial or archaeal, are generally considered autotrophs. While AOB fix CO_2_ via the Calvin–Benson–Bassham cycle (Arp *et al.*, 2007), AOA are thought to assimilate 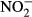 via a modified version of the 3-hydroxypropionate-4-hydroxybutyrate pathway (Berg *et al.*, 2010). However, many autotrophs are able to supplement their inorganic carbon metabolism by organic compounds that are either pathway intermediates or otherwise readily incorporated into their metabolic pathways. The capacity for mixotrophy has been proposed as an important attribute for soil AOA (Prosser & Nicol, 2012). While this trait may provide a competitive advantage to AOA over AOB in the environment, the hypothesis is almost exclusively fuelled by observations of *N. viennensis*. Pyruvate enables *N. viennensis* to grow approximately 10 times faster than in inorganic medium alone, with pyruvate-C assimilated into biomass (Tourna *et al.*, 2011). Furthermore, the marine archaeon *N. maritimus* can grow autotrophically, but growth is stimulated by the presence of pyruvate and α-ketoglutarate (Stahl & de la Torre, 2012). AOA are thought to operate an incomplete tricarboxylic acid (TCA) cycle in which pyruvate and α-ketoglutarate are intermediates (Walker *et al.*, 2010).

*Nitrosotalea devanaterra* is an obligately acidophilic ammonia oxidiser that grows by converting ammonia into nitrite within the pH range 4.0–5.5 (Lehtovirta-Morley *et al.*, 2011). *Nitrosotalea* is an abundant, globally distributed genus of AOA found in acidic soils (Gubry-Rangin *et al.*, 2011). Thirty per cent of the world's soils are considered acidic (pH < 5.5; von Uexküll & Mutert, 1995), and ammonia oxidation in low pH soils is dominated by AOA, rather than AOB (Gubry-Rangin *et al.*, 2010; Lehtovirta-Morley *et al.*, 2011). It is therefore of great interest to identify factors affecting the physiology and activity of *N. devanaterra*-like strains. *Nitrosotalea devanaterra* is the first and, currently, the only cultivated obligately acidophilic ammonia oxidiser and the model organism for studying ammonia oxidation in low pH ecosystems (Lehtovirta-Morley *et al.*, 2011).

pH plays a major role in cell physiology and moderates the effect of organic compounds. Organic acids can become toxic under acidic conditions, as small protonated acids can pass readily through the cell membrane (Alexander *et al.*, 1987; Kitko *et al.*, 2009). Upon entering the cytoplasm, dissociation of the proton results in acidification of the cytoplasm and loss of proton motive force (Salmond *et al.*, 1984). The inability of the cell to control transmembrane transport of organic acids can render acidophilic organisms vulnerable. The capability to consume organic compounds may alleviate the toxic effects of these compounds and thus make organisms less susceptible to toxicity of organic acids. This has been suggested as a reason why the most extreme acidophiles are heterotrophs; for example, the acidophile *Picrophilus* sp. which grows at an optimum pH of 1 is an obligate heterotroph (Baker-Austin & Dopson, 2007). Conversely, autotrophic acidophiles are generally adapted to oligotrophic environments and fail to respond rapidly to organic acids (Ñancucheo & Johnson, 2010). Toxicity of organic acids has been reported in several autotrophic acidophiles, including several species of *Acidithiobacillus* and *Leptospirillum ferriphilum* (Alexander *et al.*, 1987; Aston *et al.*, 2009; Ñancucheo & Johnson, 2010). In addition, organic acids are inhibitory to some acidophilic heterotrophs; *Acidiphilium* strains typically grown with low concentration of organics are inhibited by lactate and succinate (Kishimoto *et al.*, 1990).

Here, we report isolation of two strains of acidophilic soil AOA belonging to the *Nitrosotalea* genus. *Nitrosotalea devanaterra* Nd1 was isolated from a previously described enrichment culture originating from an acidic agricultural soil in Scotland (Lehtovirta-Morley *et al.*, 2011), while *Nitrosotalea* sp. Nd2 was cultivated from an acidic paddy field soil in China. The effects of pH and temperature were investigated on the two strains. In addition, the capacity for mixotrophy was examined by supplying cultures with intermediates of the TCA cycle at micromolar concentrations.

## Materials and methods

### Enrichment culture conditions

*Nitrosotalea devanaterra* Nd1 was originally enriched from an agricultural soil (pH 4.5) sampled from the Craibstone Estate, Scotland's Rural College (SRUC), Aberdeen, as previously described (Lehtovirta-Morley *et al.*, 2011). The field site is subject to an 8-year crop rotation cycle as described in detail by Kemp *et al*. (1992) and experiences a mean annual temperature of 8.3 °C. Enrichment cultures for additional acidophilic ammonia oxidisers were established using an acid sulphate paddy soil (pH 4.7) from Taishan County, Guangdong Province in Southern China (latitude/longitude: 22.11 N/112.81 E) and experiences a mean annual temperature of 21.8 °C. Double rice cropping has been practiced since 2003 where urea is used as a nitrogen fertilizer, with the paddy field receiving four applications per year, averaging 150 kg N ha^−1^ for early rice and 150 kg N ha^−1^ for later rice.

Enrichment cultures were established in triplicate as previously described (Lehtovirta-Morley *et al.*, 2011). Briefly, 1% soil inoculum was introduced into sterile mineral salts medium (pH 4.7) containing 500 μM NH_4_Cl, and enrichment cultures were incubated in darkness at 28 and 37 °C without shaking. Growth was monitored by measuring nitrite production. Both cultures were maintained routinely by transferring 2% mid-exponential culture into fresh mineral salts medium at pH 5.0. *Nitrosotalea devanaterra* Nd1 was maintained at 25 °C and *Nitrosotalea* sp. Nd2 at 37 °C.

The sensitivity of culturable bacterial contaminants to antibiotics in initial enrichment cultures was tested by adding sterile paper discs containing antibiotics to cultures spread-plated on 10% tryptone soy agar (TSA) plates. Antibiotics (ampicillin, carbenicillin, cephalexin, mecillinam, bacitracin, gentamycin, kanamycin, clindamycin, chloramphenicol, tetracycline, erythromycin, nalidixic acid) representing a range of modes of action (e.g. cell wall synthesis, translation and DNA replication) were applied as 50 μg antibiotic disc^−1^, except nalidixic acid which was used at a concentration of 15 μg disc^−1^. Inhibitory antibiotics identified from these initial screens were added to sterile liquid medium (50 mg L^−1^ for each antibiotic except nalidixic acid which was used at a concentration of 15 mg L^−1^) before inoculating with the enrichment cultures. Co-cultivated bacteria were successfully removed from the *Nitrosotalea* sp. Nd2 culture by application of 50 mg L^−1^ kanamycin. However, contaminants could not be eliminated from the *N. devanaterra* Nd1 culture with individual or combinations of antibiotics. Bacteria were therefore excluded on the basis of their comparatively larger cell size by filtration (0.45 μm pore-size) of the *N. devanaterra* Nd1 enrichment culture directly into medium containing 50 mg L^−1^ kanamycin. Neither kanamycin treatment nor filtration alone was sufficient to remove all bacteria. Purity was confirmed and monitored by plating on 10% TSA, microscopy and a lack of PCR amplification of bacterial 16S rRNA genes.

All physiology experiments were conducted in triplicate at 25 °C for *N. devanaterra* Nd1 and at 37 °C for *Nitrosotalea* sp. Nd2 unless specified otherwise. The effects of selected organic acids were determined in the presence of 100 μM of each compound. pH of the medium was adjusted to 5.0 after the addition of organic acids and was monitored throughout the experiments. Stimulation and inhibition by organic acids were expressed as the percentage increase/decrease in total nitrite concentration or specific growth rate and calculated as (treatment − inorganic control)/inorganic control. Specific growth rates was calculated by fitting a slope according to the equation: μ = (ln*N*_1_ − ln*N*_0_)/(*t*_1_ − *t*_0_), where μ represents the specific growth rate, (ln*N*_1_ − ln*N*_0_) the change in natural logarithms of nitrite concentration and (*t*_1_ − *t*_0_) the change in time. At least four time points were used for each growth rate calculation.

### Colorimetric determination of inorganic nitrogen

Ammonia and nitrite were measured colorimetrically using a 96-well plate format. Ammonia concentration was determined by the indophenol method described by Kandeler & Gerber (1988). Nitrite was measured by diazotising and coupling with Griess reagent (Shinn, 1941). Duplicate standards ranged between 50 and 500 μM NH_4_Cl and 0.781 and 50 μM NaNO_2_ for ammonia and nitrite assays, respectively. Absorbance was recorded at 620 and 540 nm, respectively, using an Infinite F50® microplate reader (Labtech, Uckfield, UK).

### Nucleic acid extraction and PCR amplification

DNA was extracted according to Tourna *et al*. (2010) with modifications described by Lehtovirta-Morley *et al*. (2011). Briefly, cultures were centrifuged, and pellets were bead-beaten in SDS-based buffer and phenol : chloroform : isoamyl alcohol mixture (25 : 24 : 1) After treating the aqueous phase with chloroform : isoamyl alcohol (24 : 1), DNA was precipitated in the presence of linear acrylamide and PEG6000. DNA was washed with 70% ethanol, dried and re-suspended in dH_2_O.

Bacteria-specific PCR was performed to monitor purity of cultures using two different primer sets targeting the bacterial 16S rRNA gene. Primers 27F and a revised version of 1492R were used together, and the second primer set consisted of P1 and P2 (Table 1). Archaeal *amoA* and 16S rRNA genes were amplified for sequencing and phylogenetic analysis from *Nitrosotalea* sp. Nd2 using primer sets 23F and 616R, and A109F and 1492R, respectively (Table 1). The composition of PCR reagent mixtures and cycling conditions have been described previously (Nicol *et al.*, 2008). PCR-amplified *Nitrosotalea* sp. Nd2 16S RNA and *amoA* gene sequences were sequenced along both strands and have been deposited in GenBank with accession numbers KJ540205 and KJ540206, respectively. *Nitrosotalea devanaterra* Nd1 16S rRNA gene and *amoA* gene sequences used for the phylogenetic analysis were sequenced previously (Lehtovirta-Morley *et al.*, 2011) and have accession numbers JN227488 and JN227489, respectively.

**Table 1:** Primers used in this study

Primer	Target organism	Gene	Sequence 5′-3′	Reference
P1	Bacteria	16S rRNA	CCTACGGGAGGCAGCAG	Muyzer *et al*. (1993)
P2	Bacteria	16S rRNA	ATTACCGCGGCTGCTGG	Muyzer *et al*. (1993)
27F	Bacteria	16S rRNA	AGAGTTTGATCCTGGCTCAG	Lane (1991)
1492R	Universal	16S rRNA	GYYACCTTGTTACGACCT	Nicol *et al*. (2008)
A109F	Archaea	16S rRNA	ACKGCTCAGTAACACGT	Großkopf *et al*. (1998)
23F	Archaea	*amoA*	ATGGTCTGGCTWAGACG	Tourna *et al*. (2008)
616R	Archaea	*amoA*	GCCATCCATCTGTATGTCCA	Tourna *et al*. (2008)

### Phylogenetic analysis

*Nitrosotalea* 16S rRNA gene and *amoA* gene sequences were aligned against sequences from cultured organisms and selected environmental clones using ClustalW implemented in bioedit Sequence Alignment Editor (Hall, 1999) before removing regions of ambiguous alignment. Phylogenetic analyses were performed using General Time Reversible-corrected maximum-likelihood (phyml; Guindon & Gascuel, 2003), parsimony (mega5; Tamura *et al.*, 2011) and Tamura's three-parameter pairwise distance analysis (mega5) for 16S rRNA gene analysis, and Jones–Taylor–Thornton (JTT)-corrected maximum-likelihood (phyml), parsimony (mega5) and JTT-corrected pairwise distance (mega5) analyses of inferred translated amino acid sequences for *amoA* genes. Where appropriate, analyses used estimated variable sites only with gamma-distributed site variation, and bootstrap support for all methods was calculated 1000 times.

### Cell enumeration

Total cell concentration was determined in 1 mL samples, fixed by addition of formaldehyde (final concentration 5%; v/v) and stored at 4 °C until enumeration. Thirty microlitres of 200 μg mL^−1^ DAPI (4,6-diamidino-2-phenylindole) was added to 1 mL of fixed cells, and samples were incubated for 5 min in the dark. Stained cells were placed onto a Cyclopore 0.22-μm pore-size black polycarbonate filter (Sigma-Aldrich, Gillingham, UK) by applying a vacuum in a standard filtration set-up and dried filters were mounted on glass slides with immersion oil and a cover slip. Cells were imaged using an Olympus BX61 fluorescence microscope equipped with a U-MWU2 fluorescence mirror unit (Olympus, Southend-on-Sea, UK). Automated cell counts were performed using imagej software (Schneider *et al.*, 2012). Five fields of view were counted on each filter. Depending on the growth stage, this equated to 10–500 cells per sample.

### Electron microscopy

Cells for both scanning and transmission electron microscopy (TEM) were harvested from an exponential culture and fixed in 4% (v/v) glutaraldehyde in phosphate-buffered saline. For scanning electron microscopy, cells were allowed to adhere to glass coverslips coated with poly-l-lysine. Slides were then rinsed with dH_2_O and postfixed with 1% OsO_4_. After washing with dH_2_O, specimens were dehydrated through a graded series of increasing ethanol concentrations (from 50% to 100%) and critical-point dried with liquid CO_2_. Samples were sputtered with Au and examined using an Eva MA 10 scanning electron microscope (Carl Zeiss, Cambridge, UK).

TEM was performed by postfixing cells with 1% OsO_4_, followed by dehydration via a graded ethanol series. Cells were transferred to propylene oxide and embedded in resin. Ultrathin sections were generated using a Leica UC6 ultramicrotome (Leica, Milton Keynes, UK), stained with uranyl acetate and lead citrate and examined using a JEM-1400 Plus transmission electron microscope (JEOL, Welwyn Garden City, UK) equipped with an UltraVUE camera (AMT, Bury St. Edmunds, UK).

### Prediction of permeability properties of organic acids

Permeability of compounds was estimated both by molecular polar surface area and the predicted concentration of protonated form of each compound. Molecular polar surface area was calculated for the studied organic acids as described by Ertl *et al*. (2000). This method is based on tabulated contributions of atoms to the polar surface area based on their bonding patterns. This value was only calculated for the form of compounds where all carboxyl groups are protonated. The threshold for permeability is considered < 60 Å^2^. If any carboxyl groups are in their dissociated form, the molecule will have a negative charge and is impermeable. Concentrations of protonated and nonprotonated forms of the compounds were calculated as follows: for pyruvate, using Henderson–Hasselbach equation pH = pK_a_ + log_10_([A^−^]/[HA]); for all other compounds except citrate using equation αH_2_A = [H^+^]^2^/([H^+^]^2^ + [H^+^]K_a1_ + K_a1_K_a2_), and for citrate using equation αH_3_A = [H^+^]^3^/([H^+^]^3^ + [H^+^]^2^K_a1_ + [H^+^]K_a1_K_a2_ + K_a1_K_a2_K_a3_), where K_a_ values are dissociation constants, and αH_2_A and αH_3_A are alpha fractions containing the protonated, that is the permeable form of the compound. It is necessary to use different equations because pyruvate has one, citrate has three, and all other compounds have two carboxyl groups.

### Statistical analyses

Statistical tests were performed in sigmaplot v12 (Systat Software). Normality and homogeneity of variance were tested with Kolmogorov–Smirnov and Levene's tests, respectively. Raw data (nitrite yield, growth rate and cell counts) were subjected to anova followed by Holm–Sidak test.

## Results

### Isolation of AOA

*Nitrosotalea devanaterra* Nd1 was maintained in an enrichment culture containing *Burkholderia* sp. for 3 years. Bacterial contaminants were removed by filtration of the culture through 0.45-μm pore-sized filters into medium containing 50 mg L^−1^ kanamycin, but ammonia oxidation and growth were not detected in this pure culture of *N. devanaterra* in mineral salts medium. A number of other antibiotics were tested but were not suitable. Nalidixic acid, tetracycline and chloramphenicol inhibited many strains of co-cultivated bacteria but inhibited nitrification. In addition, ampicillin, mecillinam and carbenicillin inhibited a few strains of bacteria on plates, but not in liquid culture, either alone or in combinations with other antibiotics. Attempts to restore growth of *N. devanaterra* Nd1 were made by supplementing medium with an array of organic compounds, including organic acids, amino acids, yeast extract and vitamins, but none enabled growth (Table S2). However, both filtered ‘spent’ medium (obtained after incubation of the mixed culture for 2 days and passed through a 0.22-μm filter) or supplementation of fresh medium with 80 mg L^−1^ casamino acids restored growth and ammonia oxidation activity by *N. devanaterra* Nd1. Although this initially suggested that *N. devanaterra* Nd1 may depend on organic supplements for growth, after approximately 10 subcultures, *N. devanaterra* Nd1 was able to grow autotrophically in unsupplemented mineral salts medium, using ammonia as its sole energy source and bicarbonate as the carbon source.

**Fig 1:**
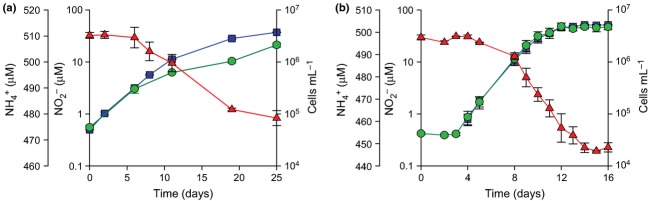
Growth and stoichiometric ammonia oxidation to nitrite by two *Nitrosotalea* isolates. (a) *Nitrosotalea devanaterra* Nd1, (b) *Nitrosotalea* sp. Nd2. Red triangles = [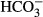], blue squares = [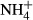], green circles = cell abundance. Error bars represent the standard error of the mean of triplicate cultures. Nitrite was below the detection limit until day 4 in *Nitrosotalea* sp. Nd2 cultures.

*Nitrosotalea* sp. Nd2 was enriched from a Chinese acidic paddy field soil by cultivation at 37 °C. A highly enriched AOA culture was obtained after several months of serial transfer using the same approach as that for *N. devanaterra* Nd1. Isolation was accomplished by addition of 50 mg L^−1^ kanamycin to inhibit the co-cultured bacteria and, unlike *N. devanaterra*, *Nitrosotalea* sp. Nd2 was able to grow autotrophically immediately after isolation. Conversion of ammonia to nitrite was stoichiometric in both isolates of *Nitrosotalea* (Fig. 1).

### Phylogeny

**Fig 2:**
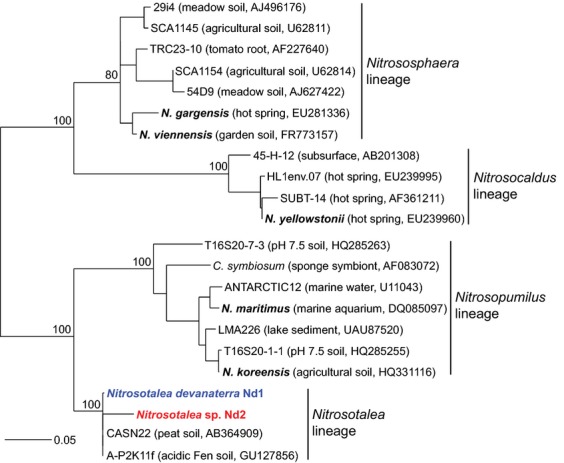
Maximum-likelihood phylogenetic analysis of 16S rRNA genes of *Nitrosotalea devanaterra* Nd1 and *Nitrosotalea* sp. Nd2 with sequences from other cultivated AOA (names in bold) and cloned environmental sequences placed with in four major AOA lineages as described by Pester *et al*. (2012) (assuming genera-level congruence between 16S rRNA gene- and *amoA* gene-defined phylogenies). Analyses were performed on 597 unambiguously aligned positions and values at major nodes represent the most conservative bootstrap support from three methods of analysis (ML, parsimony and distance). The scale bar represents 0.05 changes per nucleotide position.

Despite originating from different soils separated by considerable geographical distance, the two strains share 98.9% 16S rRNA gene and 95.1% *amoA* gene sequence identity. The novel AOA isolate from paddy field soil is phylogenetically affiliated with the *Nitrosotalea* cluster (Fig. 2, Fig. S2; Pester *et al.*, 2012), which is an abundant taxon in acidic soils globally (Gubry-Rangin *et al.*, 2011). Although the two strains have a greater 16S rRNA gene similarity than the recommended species cut-off of 97.5% at the 16S rRNA gene level (Konstantinidis & Tiedje, 2005), the name *Nitrosotalea* sp. Nd2 is proposed for this organism, in contrast to the type strain *N. devanaterra* Nd1, due to moderate differences *amoA* gene sequence identity and a lack of genome data for comparison.

### Morphology

**Fig 3:**
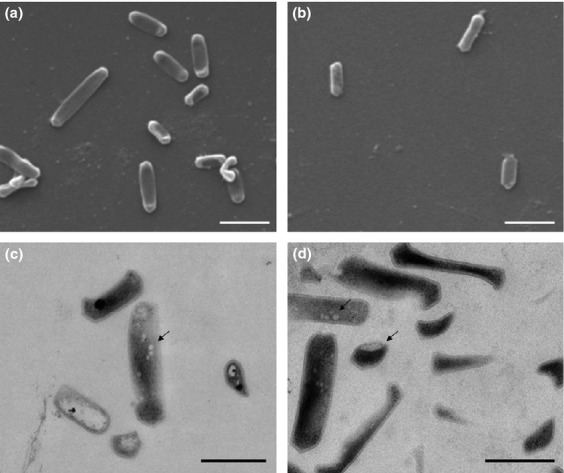
Scanning (a and b) and transmission (c and d) electron micrographs of *Nitrosotalea devanaterra* Nd1 (a and c) and *Nitrosotalea* sp. Nd2 (b and d). Arrows indicate subcellular particles. Scale bars are 1 μm (a and b) and 0.5 μm (c and d).

Both *N. devanaterra* isolates are morphologically indistinguishable and are small rods, typically 0.5–1 μm in length (Fig. 3). Both display a slightly angular appearance with electron-dense poles. Interestingly, during exponential growth, *N. devanaterra* cells occasionally became very elongated, reaching up to 2 μm in length (Fig. 3a). Intracellular compartmentalisation was evident in TEM. However, the membrane-bound intracellular compartment previously reported in *Nitrosopumilus*-like species (Jung *et al.*, 2011; Yan *et al.*, 2012) was not visible in the *Nitrosotalea* strains. Both strains contained apparent subcellular particles and inclusion bodies (indicated by arrows in Fig. 3), which may be storage granules.

### Physiology of AOA isolates

Despite being closely related, the two *Nitrosotalea* isolates exhibit different physiologies. Specific growth rates of *N. devanaterra* Nd1 and *Nitrosotalea* sp. Nd2 were strikingly different; *Nitrosotalea* sp. Nd2 grows approximately twice as fast as *N. devanaterra* Nd1 [μ ≈ 0.60 (SE 0.01) day^−1^ and 0.29 (SE 0.01) day^−1^, respectively]. Cell yields of *N. devanaterra* Nd1 and *Nitrosotalea* sp. Nd2 cultures were similar, with 6.45 × 10^4^ (SE 5.1 × 10^2^) cells (μM 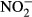)^−1^ and 7.0 × 10^4^ (SE 8.2 × 10^3^ cells (μM 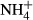)^−1^, respectively. Cell activities were also similar: 71.8 (SE 4.3) amol 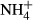 cell^−1^ h^−1^ for *N. devanaterra* Nd1 and 64.5 (SE 4.4) amol 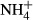 cell^−1^ h^−1^ for *Nitrosotalea* sp. Nd2. Assuming a similar protein content to that of *N. maritimus*, based on their similar size and morphology, these activities can be alternatively expressed as approximately 7.0 (SE 0.42) and 6.3 (SE 0.43) μmol 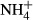 mg protein^−1^ h^−1^ for *N. devanaterra* Nd1 and *Nitrosotalea* sp. Nd2, respectively. Activity and yield of *N. devanaterra* Nd1 were different in pure cultures and enrichments, most likely due to different enumeration methods (qPCR vs. direct cell counts). Discrepancy between the two methods has been previously reported in a pure culture of *N. maritimus* (Nakagawa & Stahl, 2013). Alternatively, it is possible that this difference is a result from the lack of bacterial metabolites after isolation. Growth of both cultures was affected by nitrite accumulation and subsequent toxicity from nitrous acid, and growth terminated before all ammonium was depleted. Maximum nitrite concentration at the onset of stationary phase was strongly pH-dependent, as previously reported for the enrichment culture of *N. devanaterra* Nd1 (Lehtovirta-Morley *et al.*, 2011), and *N. devanaterra* Nd1 and *Nitrosotalea* sp. Nd2 were inhibited at 0.91–3.50 μM and 1.61–5.70 μM HNO_2_, respectively (assumed from the Henderson–Hasselbach equation). Under typical growth conditions (pH 5), final nitrite concentration was ∼50 μM and the highest values recorded were 49.0 (SE 1.2) μM for *N. devanaterra* Nd1 and 54.3 (SE 0.1) μM for *Nitrosotalea* sp. Nd2. The highest nitrite concentrations in inorganically grown cultures were 101 (SE 0.9) μM for *N. devanaterra* Nd1 (pH 5.6) and 112 (SE 0.8) μM for *Nitrosotalea* sp. Nd2 (pH 6.1).

**Fig 4:**
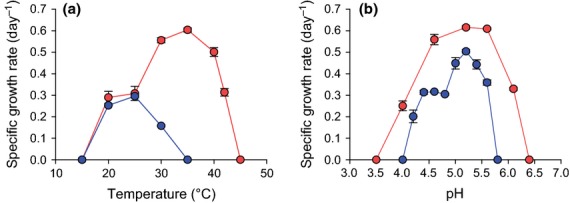
The effect of temperature (a) and pH (b) on the specific growth rate of *Nitrosotalea devanaterra* isolates. In a, the left *y*-axis represents *N. devanaterra* Nd1, the right *y*-axis *Nitrosotalea* sp. Nd2. Blue circles = *N. devanaterra* Nd1, red circles = *Nitrosotalea* sp. Nd2. Error bars represent the standard error of the mean of triplicate cultures.

Temperature preference differed between the *Nitrosotalea* strains (Fig. 4a). Optimal growth of *N. devanaterra* Nd1 was observed at 25 °C [μ = 0.29 (SE 0.01) day^−1^] and growth occurred within the range of 20–30 °C. In contrast, *Nitrosotalea* sp. Nd2 grew optimally at 35 °C [0.60 (SE 0.01) day^−1^], a temperature that completely inhibited growth of *N. devanaterra* Nd1. In addition, *Nitrosotalea* sp. Nd2 had a wider temperature range (20–42 °C) than *N. devanaterra* Nd1. Interestingly, the specific growth rate of *Nitrosotalea* sp. Nd2 was lower at 25 °C and comparable to that of *N. devanaterra* Nd1 under the same conditions. Preference for low pH is the defining feature of currently recognised members of the genus *Nitrosotalea*. *Nitrosotalea* sp. Nd2 is an obligate acidophile with a slightly broader pH range for growth (4.0–6.1; Fig. 4b) than *N. devanaterra* Nd1 (4.2–5.6).

### Effect of organic acids on acidophilic ammonia oxidisers

**Fig 5:**
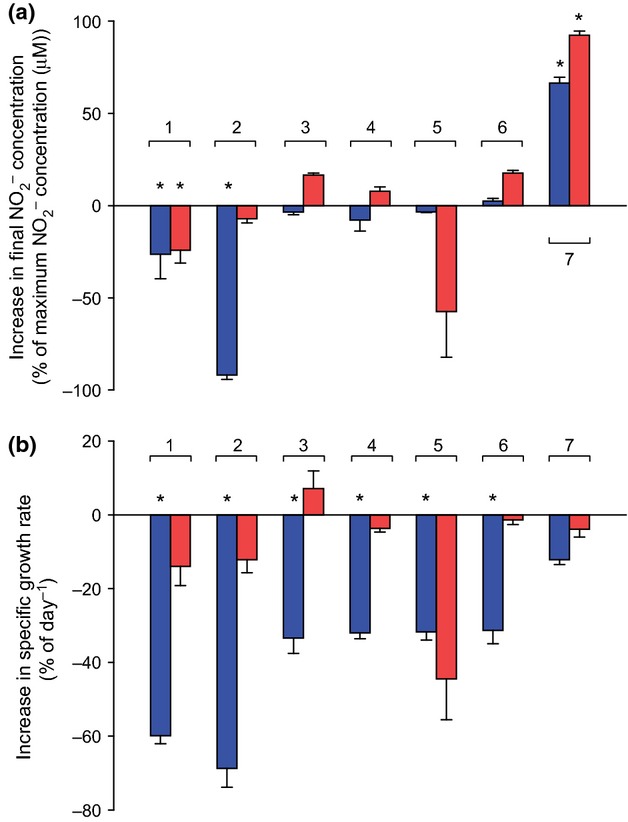
Effects of addition of 100 μM TCA cycle intermediates on nitrite yield (a) and specific growth rate (b) of *Nitrosotalea* isolates expressed as a percentage compared with inorganically grown control. Blue bars = *Nitrosotalea devanaterra* Nd1, red bars = *Nitrosotalea* sp. Nd2. 1 = pyruvate, 2 = citrate, 3 = α-ketoglutarate, 4 = succinate, 5 = fumarate, 6 = malate, 7 = oxaloacetate. Error bars represent the standard error of the mean of triplicate cultures. Asterisk indicates statistically significant difference to inorganically grown control (*P *<* *0.05).

Surprisingly, many TCA cycle intermediates inhibited *N. devanaterra* Nd1 and *Nitrosotalea* sp. Nd2. Pyruvate (100 μM) decreased the cell yield of both organisms (Fig. 5 and Fig. S2) and reduced the final nitrite concentration, being 26% lower in *N. devanaterra* Nd1 cultures [22.0 (SE 1.2) vs. 36.4 (SE 0.4) μM]. In addition, cell yield of *N. devanaterra* Nd1 was reduced (45%) in the presence of pyruvate (Fig. S2a). Exposure of *Nitrosotalea* sp. Nd2 to pyruvate reduced nitrite and cell yields to 24% and 34%, respectively. Specific growth rate of *N. devanaterra* Nd1 was reduced by pyruvate from 0.29 (SE 0.01) day^−1^ to 0.12 (SE 0.01) day^−1^. Growth rate of *Nitrosotalea* sp. Nd2 was not reduced significantly in the presence of pyruvate.

The *Nitrosotalea* strains responded differently to many organic acids, underlining their distinct physiologies. Apart from oxaloacetate, all the tested TCA cycle intermediates significantly decreased the specific growth rate of *N. devanaterra* Nd1 (Fig. 5b), while none of the tested compounds had a significant effect on the specific growth rate of *Nitrosotalea* sp. Nd2. Citrate strongly inhibited *N. devanaterra* Nd1, resulting in decreases to 69% and 93% in nitrite and biomass yield, but had no effect on *Nitrosotalea* sp. Nd2. In contrast, fumarate inhibited *Nitrosotalea* sp. Nd2 but not *N. devanaterra* Nd1.

Oxaloacetate was the only tested compound to stimulate both *Nitrosotalea* strains significantly. While specific growth rates remained unchanged, both nitrite and cell yields increased in the presence of oxaloacetate. Cell abundance was 60% and 117% higher for *N. devanaterra* Nd1 and *Nitrosotalea* sp. Nd2, respectively, while maximum nitrite concentrations were 66% and 92% greater. Generally the yield of acidophilic AOA is dictated by pH and the consequent concentration of nitrous acid. However, the apparent advantage granted by oxaloacetate may not be due to buffering effects or pH changes. The final pH was 0.09 and 0.16 units higher for *N. devanaterra* Nd1 and *Nitrosotalea* sp. Nd2 cultures, respectively, in oxaloacetate-supplemented cultures compared with the control. The final concentration of nitrous acid was 3.63 (SE ± 0.07) and 6.26 (SE 0.07 μM) for *N. devanaterra* Nd1 and *Nitrosotalea* sp. Nd2, respectively, which indicates slightly higher tolerance than in inorganically grown cultures.

Although oxaloacetate increased nitrite and cell concentrations, proportional changes were similar (Fig. S2b) and yield expressed as cells generated (1 μM 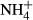 produced)^−1^ was not significantly different between any of the treatments with organic acids or the control. This suggests that under the tested conditions, *Nitrosotalea* strains derive their energy for growth by oxidising ammonia rather than organic compounds.

Membrane permeability of organic acids could play a critical role in their toxicity in low pH. Permeability estimated as the molecular polar surface area was calculated for the studied compounds (Table S3). Only fully protonated compounds can be permeable, so concentration of this form was also calculated for each compound. Only pyruvate was highly permeable, with a value of 54.4 Å^2^ (< 60 Å^2^ is considered a threshold for permeability).

## Discussion

This study describes isolation and characterisation of two acidophilic AOA: *N. devanaterra* Nd1 and *Nitrosotalea* sp. Nd2. Interestingly, while the two AOA isolates are very closely related in terms of 16S rRNA gene and *amoA* gene phylogenies, their physiologies differed with respect to optimal growth temperature, specific growth rate and response to organic acids. 16S rRNA gene similarity is used as the foundation in taxonomy and molecular ecology studies. This therefore highlights the dangers in assuming strong links between 16S rRNA gene and functional gene phylogeny and function at the levels of sequence resolution achievable using such genes and typically employed in molecular studies. Despite high identity in 16S rRNA gene and *amoA* genes, these two strains differed in physiological characteristics that are likely to be of ecological importance. In addition, it is interesting that such (phylogenetically) closely related strains were isolated from ecosystems separated by a vast distance. This may indicate the selection by the growth medium and cultivation conditions employed for *N. devanaterra*-like phylotypes rather than other ammonia oxidisers present in acidic soils.

Contrasting temperature preferences of the *Nitrosotalea* isolates may reflect the origins of the organisms; the mean annual temperature of 21.8 °C in the Chinese paddy field soil was considerably higher than that of the Scottish agricultural soil (8.3 °C). The optimal growth temperature (35 °C) of *Nitrosotalea* sp. Nd2 is also similar to cultivated *Nitrososphaera gargensis* and *N. viennensis* (Hatzenpichler *et al.*, 2008; Tourna *et al.*, 2011).

Although cell yields of the *Nitrosotalea* strains were similar to each other, they were substantially lower than typically observed for AOA [4 × 10^5^ cells (μM 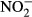)^−1^ for *N. maritimus*]. Furthermore, cell activities of both *Nitrosotalea* were approximately eightfold lower than values reported for *N. maritimus* (51.9 μmol 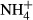 mg protein^−1^ h^−1^). This difference may reflect a higher energy expenditure required for intracellular pH regulation in acidic conditions. Interestingly, the specific growth rate of *Nitrosotalea* sp. Nd2 is comparable to that of *N. maritimus* (maximum reported μ = 0.64 day^−1^; Martens-Habbena *et al.*, 2009) and considerably higher than that of *N. devanaterra* Nd1.

In contrast to neutrophilic AOA, growth of both *Nitrosotalea* isolates was inhibited by nitrite accumulation and ceased prior to substrate depletion. As maximum nitrite yield in *Nitrosotalea* cultures depended strongly on the cultivation pH, nitrous acid rather than nitrite is presumably mediating this toxicity. The amount of nitrous acid accumulated by the *Nitrosotalea* strains is equivalent to > 13 mM nitrite at pH 7.5. Little is known about nitrite/nitrous acid toxicity in other AOA, but it is plausible that *Nitrosotalea* strains are no more sensitive to nitrous acid than any neutrophilic AOA. In terms of ecology, this suggests that AOA must maintain a tight partnership with nitrite oxidisers in acidic soils. Although nitrite rarely accumulates in soils (Paul & Clarke, 1989), nitrite oxidisers may play a more crucial role for ammonia oxidisers in acidic rather than neutral soils. In soil microcosms where *N. devanaterra* drives nitrification, close to 100 μg nitrate–N g^−1^ soil was accumulated without apparent toxicity (Lehtovirta-Morley *et al.*, 2011), indicating that nitrate is likely to be less toxic than nitrite is in these ecosystems.

Inhibition of growth by organic acids may be caused by interference with intracellular pH regulation. Small, nonpolar molecules can readily penetrate membranes when protonated, and dissociation within the cytoplasm leads to acidification and uncoupling of energy-generating mechanisms (Baker-Austin & Dopson, 2007). Molecular polar surface area was used as a predictor of membrane permeability (Ertl *et al.*, 2000). Pyruvate was the only highly membrane-permeable compound in this study (Table S3). However, in a neutral pH environment, metabolism of pyruvate may necessitate active transport, as a very small proportion will exist in the membrane-permeable, protonated form. For example, even at the highest reported concentration (10 mM) for *N. viennensis*, only 0.1 μM pyruvate is protonated. In contrast, 0.32 μM pyruvate was present in the protonated form in the experiments with both *Nitrosotalea* strains, equivalent to ∼32 mM pyruvate in the typical growth conditions of *N. viennensis*.

The concentrations of organic acids tested in this study were micromolar to mimic concentrations typical of rhizosphere soil (Jones, 1998). In soil ecosystems, readily assimilable organic compounds are in their highest concentrations in the rhizosphere (Cieśliński *et al*., 1998). Although *N. viennensis* grows in medium containing a wide range of pyruvate concentrations (0.001–10 mM), stimulation was greatest at 100 μM and did not increase at higher pyruvate concentrations. No information has been released about the stimulatory concentrations of organic acids in *N. maritimus*. If mixotrophy is a genuine metabolic feature of AOA, these data indicate that there is variation in preference for the organic substrates utilised. AOA belonging to *Nitrososphaera* and *Nitrosopumilus* genera may preferentially reside in the rhizospheric soil, whereas the opposite would be true for species belonging to *Nitrosotalea*. Alternatively, in soil *Nitrosotalea* strains may benefit from proximity of heterotrophs which rapidly degrade inhibitory organic acids. It remains to be seen whether AOA belonging to *Nitrosopumilus* and *Nitrososphaera* can successfully compete against heterotrophs for organics in soil. Results from previously published *in situ* studies are contradictory and thaumarchaeal/AOA abundance has been reported to be both higher (Ochsenreiter *et al.*, 2003; Dias *et al.*, 2012) and lower (Herrmann *et al.*, 2008) in bulk soil than in the rhizosphere. The differential preferences of ammonia oxidisers to organic compounds may contribute to this discrepancy and also shape ammonia oxidiser community structures.

In conclusion, this study demonstrates that *Nitrosotalea* strains can be isolated in pure culture and even closely related AOA strains do not display identical physiology Furthermore, although pyruvate is stimulatory to *N. viennensis* and *N. maritimus*, it is slightly inhibitory to *Nitrosotalea* strains. Capacity for mixotrophy, or the lack of it, may be an important contributor to niche differentiation of AOA.

## Supporting Information

Fig S1 Effects of addition of 100 μM TCA cycle intermediates on cell abundance (a) and yield normalised against  production (b).

Fig S2 Maximum-likelihood phylogenetic analysis of inferred AmoA amino acid sequences translated from *amoA* gene sequences of *Nitrosotalea devanaterra* Nd1 and *Nitrosotalea* sp.

Table S1 AOA cultures reported in literature. **Table S2.** Capacity of organic compounds to restore growth in the pure culture of *Nitrosotalea devanaterra*. **Table S3.** Properties of the examined organic acids.
