# Supplementary material for: Characterisation of terrestrial acidophilic archaeal ammonia oxidisers and their inhibition and stimulation by organic compounds
Source: FEMS Microbiol Ecol. 2014 Jul 31;89(3):542–52. doi: 10.1111/1574-6941.12353 (PMC4261999; doi:10.1111/1574-6941.12353)
Supplement: Table S1 — AOA cultures reported in literature. Table S2. Capacity of organic compounds to restore growth in the pure culture of Nitrosotalea devanaterra. Table S3. Properties of the examined organic acids. [file fem0089-0542-SD3.docx]

| **Supplementary Table 1.** AOA cultures reported in literature | | | | |
| --- | --- | --- | --- | --- |
| Organism | Strain | Origin | Reference | |
| ***Nitrosopumilus maritimus*** | **SCM1** | **Seawater aquarium** | **Könneke *et al.*** | **(2005)** |
| *Nitrosocaldus yellowstoneii* | HL72 | Hot spring | De la Torre *et al.* | (2008) |
| *Nitrososphaera gargensis* | Ga9.2 | Hot spring | Hatzenpichler *et al.* | (2008) |
| *Nitrosopumilus* sp. | SJ | Marine sediment | Park *et al.* | (2010) |
| ***Nitrososphaera viennensis*** | **EN76**, EN123 | **Garden soil** | **Tourna *et al.*** | **(2011)** |
| *Nitrosotalea devanaterra* | Nd1 | Agricultural soil | Lehtovirta-Morley *et al.* | (2011) |
| *Nitrosoarchaeum koreensis* | MY1 | Agricultural soil | Jung *et al.* | (2011) |
| *Nitrosopumilus* sp. | NM25 | Coastal sand of an eelgrass zone | Matsutani *et al.* | (2011) |
| *Nitrosopumilus* sp. | 3 strains | Marine water column | Santoro & Casciotti | (2011) |
| *Nitrosoarchaeum limnia* | SFB1 | Estuary sediment | Blainey *et al.* | (2011) |
| *Nitrosoarchaeum limnia* | BG20 | Estuary sediment | Mosier *et al.* | (2012a) |
| *Nitrosopumilus salaria* | BD31 | Estuary sediment | Mosier *et al.* | (2012b) |
| *Nitrosopumilus koreensis* | AR1 | Marine sediment | Park *et al.* | (2012a) |
| *Nitrosopumilus sediminis* | AR2 | Marine sediment | Park *et al.* | (2012b) |
| *Nitrosotenuis* sp. | 3 strains | Freshwater | French *et al.* | (2012) |
| *Nitrososphaera* sp. | JG1 | Agricultural soil | Kim *et al.* | (2012) |
| *Nitrososphaera* sp. & *Nitrososphaera*-sister lineage | >2 strains | Arctic soil | Alves *et al.* | (2013) |
| *Nitrosotenuis uzonensis* | N4 | Hot spring | Lebedeva *et al.* | (2013) |
| *Nitrosotenuis* sp. | MY2 | Agricultural soil | Jung *et al.* | (2013) |
| *Nitrososphaera* sp. | MY3 | Contaminated soil | Jung *et al.* | (2013) |
| *Nitrosotalea* sp. | CS | Acidic mine | Jung *et al.* | (2013) |

Organisms highlighted in bold have been isolated in pure culture

Alves RJ, Wanek W, Zappe A, Richter A, Svenning MM, Schleper C & Urich T (2013) Nitrification rates in Arctic soils are associated with functionally distinct populations of ammonia-oxidizing archaea. *ISME J* 7:1620-31.

Blainey PC, Mosier AC, Potanina A, Francis CA & Quake SR (2011) Genome of a low-salinity ammonia-oxidizing archaeon determined by single-cell and metagenomic analysis. *PLoS One* **22:** e16626.

de la Torre JR, Walker CB, Ingalls AE, Könneke M & Stahl DA (2008) Cultivation of a thermophilic ammonia oxidizing archaeon synthesizing crenarchaeol. *Environ Microbiol* **10:** 810-818.

French E, Kozlowski JA, Mukherjee M, Bullerjahn G & Bollmann A (2012) Ecophysiological Characterization of Ammonia-Oxidizing Archaea and Bacteria from Freshwater. Appl Environ Microbiol **78:** 5773-5780.

Hatzenpichler R, Lebedeva EV, Spieck E, Stoecker K, Richter A, Daims H &Wagner M (2008) A moderately thermophilic ammonia-oxidizing crenarchaeote from a hot spring. *Proc Natl Acad Sci USA* **105:** 2134-2139.

Jung MY, Park SJ, Min D, Kim JS, Rijpstra WIC, Sinninghe Damste JS, Kim GJ, Madsen EL & Rhee SK (2011) Enrichment and characterization of an autotrophic ammonia-oxidizing archaeon of mesophilic crenarchaeal group I.1a from an agricultural soil. *Appl Environ Microbiol* **77:** 8635–8647.

Jung MY, Well R, Min D, Giesemann A, Park SJ, Kim JG, Kim SJ & Rhee SK (2014) Isotopic signatures of N_2_O produced by ammonia-oxidizing archaea from soils. *ISME J* (in press).

Kim JG, Jung MY, Park SJ, Rijpstra WI, Sinninghe Damsté JS, Madsen EL, Min D, Kim JS, Kim GJ & Rhee SK (2012) Cultivation of a highly enriched ammonia-oxidizing archaeon of thaumarchaeotal group I.1b from an agricultural soil. *Environ Microbiol* **14:** 1528-1543.

Könneke M, Bernhard AE, de la Torre JR, Walker CB, Waterbury JB & Stahl DA (2005) Isolation of an autotrophic ammonia-oxidizing marine archaeon. *Nature* **437:** 543-546.

Lebedeva EV, Hatzenpichler R, Pelletier E, Schuster N, Hauzmayer S, Bulaev A, Grigor’eva NV, Galushko A, Schmid M, Palatinszky M, *et al.,* (2013) Enrichment and Genome Sequence of the Group I.1a Ammonia-Oxidizing Archaeon “*Ca.* Nitrosotenuis uzonensis” Representing a Clade Globally Distributed in Thermal Habitats. *PLoS ONE*  **8**: e80835.

Lehtovirta-Morley LE, Stoecker K, Vilcinskas A, Prosser JI &Nicol GW (2011) Cultivation of an obligate acidophilic ammonia oxidizer from a nitrifying acid soil. *Proc Natl Acad Sci USA* **108**: 15892-15897.

Matsutani N, Nakagawa T, Nakamura K, Takahashi R, Yoshihara K & Tokuyama T (2011) Enrichment of a novel marine ammonia-oxidizing archaeon obtained from sand of an eelgrass zone. *Microbes Environ* **26**: 23-29.

Mosier AC, Allen EE, Kim M, Ferriera S & Francis CA (2012) Genome Sequence of “*Candidatus* Nitrosoarchaeum limnia” BG20, a Low-Salinity Ammonia-Oxidizing Archaeon from the San Francisco Bay Estuary. *J Bacteriol* **194**: 2119-2120.

Mosier AC, Allen EE, Kim M, Ferriera S & Francis CA (2012) Genome sequence of "*Candidatus* Nitrosopumilus salaria" BD31, an ammonia-oxidizing archaeon from the San Francisco Bay estuary. *J Bacteriol* **194**: 2121-2122.

Park BJ, Park SJ, Yoon DN, Schouten S, Sinninghe Damsté JS & Rhee SK (2010) Cultivation of autotrophic ammonia-oxidizing archaea from marine sediments in coculture with sulfur-oxidizing bacteria. *Appl Environ Microbiol* **76**: 7575-7587.

Park SJ, Kim JG, Jung MY, Kim SJ, Cha IT, Kwon K, Lee JH & Rhee SK (2012) Draft Genome Sequence of an Ammonia-Oxidizing Archaeon, “*Candidatus* Nitrosopumilus koreensis” AR1, from Marine Sediment. *J Bacteriol* **194**: 6940-6941.

Park SJ, Kim JG, Jung MY, Kim SJ, Cha IT, Ghai R, Martín-Cuadrado AB, Rodríguez-Valera F & Rhee SK (2012) Draft Genome Sequence of an Ammonia-Oxidizing Archaeon, “*Candidatus* Nitrosopumilus sediminis” AR2, from Svalbard in the Arctic Circle. *J Bacteriol* **194**: 6948-6949.

Tourna M, Stieglmeier M, Spang A, Könneke M, Schintlmeister A, Urich T, Engel M, Schloter M, Wagner M, Richter A *et al.* (2011) *Nitrososphaera viennensis*, an ammonia oxidizing archaeon from soil. *Proc Natl Acad Sci USA* 108: 8420-8425.

Santoro AE & Casciotti KL (2011) Enrichment and characterization of ammonia-oxidizing archaea from the open ocean: phylogeny, physiology and stable isotope fractionation. *ISME J* **5:** 1796–1808.

| \| **Supplementary Table 2.** Capacity of organic compounds to restore growth in the pure culture of *N. devanaterra* \| \| \| \| --- \| --- \| --- \| \| Substrate \| Effect \| Lag phase \| \| 100 µM glutamic acid \| - \| - \| \| 100 µM glycine \| - \| - \| \| 100 µM Na pyruvate \| - \| - \| \| 100 µM Na propionate \| - \| - \| \| 100 µM Na formate \| - \| - \| \| 100 µM Na acetate \| - \| - \| \| 100 µM succinate \| - \| - \| \| 100 µM glyoxylate \| - \| - \| \| 100 µM oxaloacetate \| - \| - \| \| 100 µM α-ketoglutarate \| - \| - \| \| Vitamin solution* \| - \| - \| \| 0.001% yeast extract \| - \| - \| \| 0.08 g l^-1^ casein hydrolysate \| + \| Short (1 d) \| \| Spent media \| + \| Long (>30 d) \|   *Vitamin solution consisted of 20 mg l^-1^ biotin, 50 mg l^-1^ pyridoxamine, 50 mg l^-1^ thiamine, 50 mg l^-1^ nicotinic acid, 50 mg l^-1^ calcium pantothenate, 50 mg l^-1^ *p*-aminobenzoic acid and 10 mg l^-1^ vitamin B12.  **Supplementary Table 3.** Properties of the examined organic acids | | | | |
| --- | --- | --- | --- | --- | --- | --- | --- | --- | --- | --- | --- | --- | --- | --- | --- | --- | --- | --- | --- | --- | --- | --- | --- | --- | --- | --- | --- | --- | --- | --- | --- | --- | --- | --- | --- | --- | --- | --- | --- | --- | --- | --- | --- | --- | --- | --- | --- | --- | --- | --- | --- | --- |
| Compound | Molecular polar surface ratio (Å^2^) | pK_a_ | At pH 5.0, 100 µM organic acid | |
|  |  |  | Concentration of fully protonated form (µM) | Concentration of non-protonated form (µM) |
| Pyruvate | 54.4 | 2.50 | 0.32 | 99.68 |
| Citrate | 132.1 | 3.06, 4.76, 5.40 | 0.33 | 99.67 |
| α-ketoglutarate | 91.7 | 1.90, 4.44 | 0.017 | 99.98 |
| Succinate | 74.6 | 4.21, 5.64 | 11.66 | 88.34 |
| Fumarate | 74.6 | 3.03, 4.44 | 0.23 | 99.77 |
| Malate | 94.4 | 3.40, 5.20 | 1.52 | 98.48 |
| Oxaloacetate | 91.7 | 2.22, 3.89 | 0.012 | 99.99 |
