# Supplementary material for: Characterisation of terrestrial acidophilic archaeal ammonia oxidisers and their inhibition and stimulation by organic compounds
Source: FEMS Microbiol Ecol. 2014 Jul 31;89(3):542–52. doi: 10.1111/1574-6941.12353 (PMC4261999; doi:10.1111/1574-6941.12353)
Supplement: Supplementary file 4 — Supplementary [file fem0089-0542-SD4.docx]

**Supplementary figure legends**

**Supplementary Figure 1.** Effects of addition of 100 µM TCA cycle intermediates on cell abundance (A) and yield normalised against NO_2_^-^ production (B). Blue bars = *N. devanaterra*, green bars = *Nitrosotalea* sp. Nd2. 1 = pyruvate, 2 = citrate, 3 = α-ketoglutarate, 4 = succinate, 5 = fumarate, 6 = malate, 7 = oxaloacetate. Error bars represent the standard error of the mean of triplicate cultures. Asterisk indicates statistically significant difference to inorganically grown control (*p*<0.05).

**Supplementary Figure 2.** Maximum-likelihood phylogenetic analysis of inferred AmoA amino acid sequences translated from *amoA* gene sequences of *N. devanaterra* Nd1 and *Nitrosotalea* sp. Nd2 with sequences from other cultivated AOA (names in bold) and cloned environmental sequences placed within four major AOA lineages as described by Pester *et al.,* (2012). Analyses were performed on 162 unambiguously aligned positions and values at major nodes represent the most conservative bootstrap support from three methods of analysis (ML, parsimony and distance). The scale bar represents 0.05 changes per amino acid position.
